# Three-Dimensional CT-Based Planning for Reverse Shoulder Arthroplasty in Chronic Anterior Dislocation: Tips and Tricks

**DOI:** 10.7759/cureus.103456

**Published:** 2026-02-12

**Authors:** Georgios Tsinaslanidis, David Ensor, Georgios Mamarelis, Dimitrios Tsekes

**Affiliations:** 1 Trauma and Orthopaedics, Barking, Havering and Redbridge University Hospitals NHS Trust, London, GBR; 2 Trauma and Orthopaedics, Barts Health NHS Trust, London, GBR; 3 Trauma and Orthopaedics, Royal Free London NHS Foundation Trust, London, GBR

**Keywords:** chronic anterior shoulder dislocation, ct, preoperative planning, reverse shoulder replacement, tips and tricks

## Abstract

This case report describes the management of a patient with a chronic (18-month) fixed anterior shoulder dislocation with an anterior glenoid bony defect of <25%. This patient was wheelchair-bound, and it was important to restore a functional, pain-free shoulder to maintain the patient’s quality of life and independence. Management of this injury remains challenging and must be individualised according to patient and injury factors. The surgical team chose to treat the patient with a reverse shoulder arthroplasty, in light of the chronic nature of the injury, the patient’s comorbidities and mobility needs. The surgical dissection was expected to be challenging, and the procedure was planned using pre-operative planning software. The dissection of the humeral head was challenging due to the formation of adhesions, osteophytes inferior to the native glenoid and severe soft-tissue scarring. The humeral head was approached from an initial deltopectoral approach and extensive releases, in order to mobilise the humeral head and perform the humeral cut. In addition, three-dimensional (3D) evaluation proved invaluable for accurate assessment of this complex glenoid deformity. The glenoid bony defect was managed by orientating the eccentric glenosphere positioned anteriorly and a high-offset humeral tray, with the high-offset positioned posteriorly. This construct would accommodate and neutralise the significant forces from the soft-tissue scarring that would make the replacement prone to dislocation. This case demonstrates that good prosthetic fixation can be achieved, despite a bony defect through minor alteration of the orientation of prosthetic components.

## Introduction

The incidence of chronic anterior shoulder dislocations is rare but undoubtedly poses a challenge to the orthopaedic surgeon in determining the appropriate treatment. Rowe et al. initially classified it as greater than three weeks, and Goga et al. proposed to classify chronic dislocation after more than one week, and then with further sub-classifications [1,2]. The authors of this article considered a dislocation to be chronic if it persisted beyond 21 days post-trauma. Surgical options include reconstruction, such as the Latarjet procedure or arthroplasty, ranging from humeral head resurfacing, total anatomical shoulder arthroplasty, hemiarthroplasty and reverse shoulder arthroplasty (RSA). The choice of treatment depends on the patient’s functional demands, rotator cuff integrity and bone stock. Currently, there is no consensus on treatment options, especially in the event of bone loss [3].

This article presents a case of a chronic anterior dislocation, which was treated with a reverse shoulder replacement using three-dimensional computed tomography (3D CT)-based Blueprint pre-operative planning. This patient was wheelchair-bound and relied on his upper limbs for independent mobilisation. The surgical challenges in this case included the chronicity of the dislocation, glenoid bone loss, formation of adhesions between the humeral head and the neo-glenoid and the presence of osteophytes. This article outlines both the 3D CT-based pre-operative planning and the intraoperative techniques employed to overcome these challenges.

## Case presentation

A 70-year-old White European male presented with an anterior dislocation of the right shoulder following a fall. The initial presentation was delayed by three weeks, when his general practitioner eventually arranged an X-ray confirming the dislocation. He was first reviewed in the orthopaedic clinic six months post-injury.

His past medical history included cerebellar syndrome with ataxia, involuntary movements, poor balance and dysarthria resulting in frequent falls. Additional comorbidities included peripheral neuropathy, moderate chronic obstructive pulmonary disease, vitamin B12 deficiency and hypertension, for which he was taking ramipril. He was semi-independent at home requiring assistance from his sister, alongside formal carers, mobilising with a three-wheel walker indoors and a wheelchair outdoors. He was right hand dominant and an ex-smoker of 30 years.

**Figure 1:**
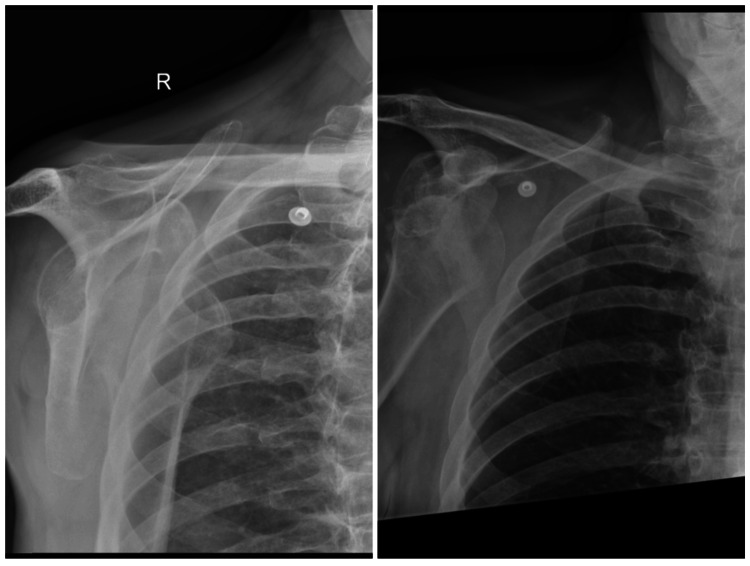
Pre-operative radiographs demonstrating chronic anterior dislocation of the right glenohumeral joint.

During his first clinic appointment, the patient reported debilitating right shoulder pain, limited movement and nocturnal discomfort that prevented sleep. He was unable to self-propel his wheelchair. Examination revealed loss of his normal shoulder contour with inferior and medial displacement of his humeral head relative to the coracoid, confirmed on X-ray (Figure 1). 

**Figure 2:**
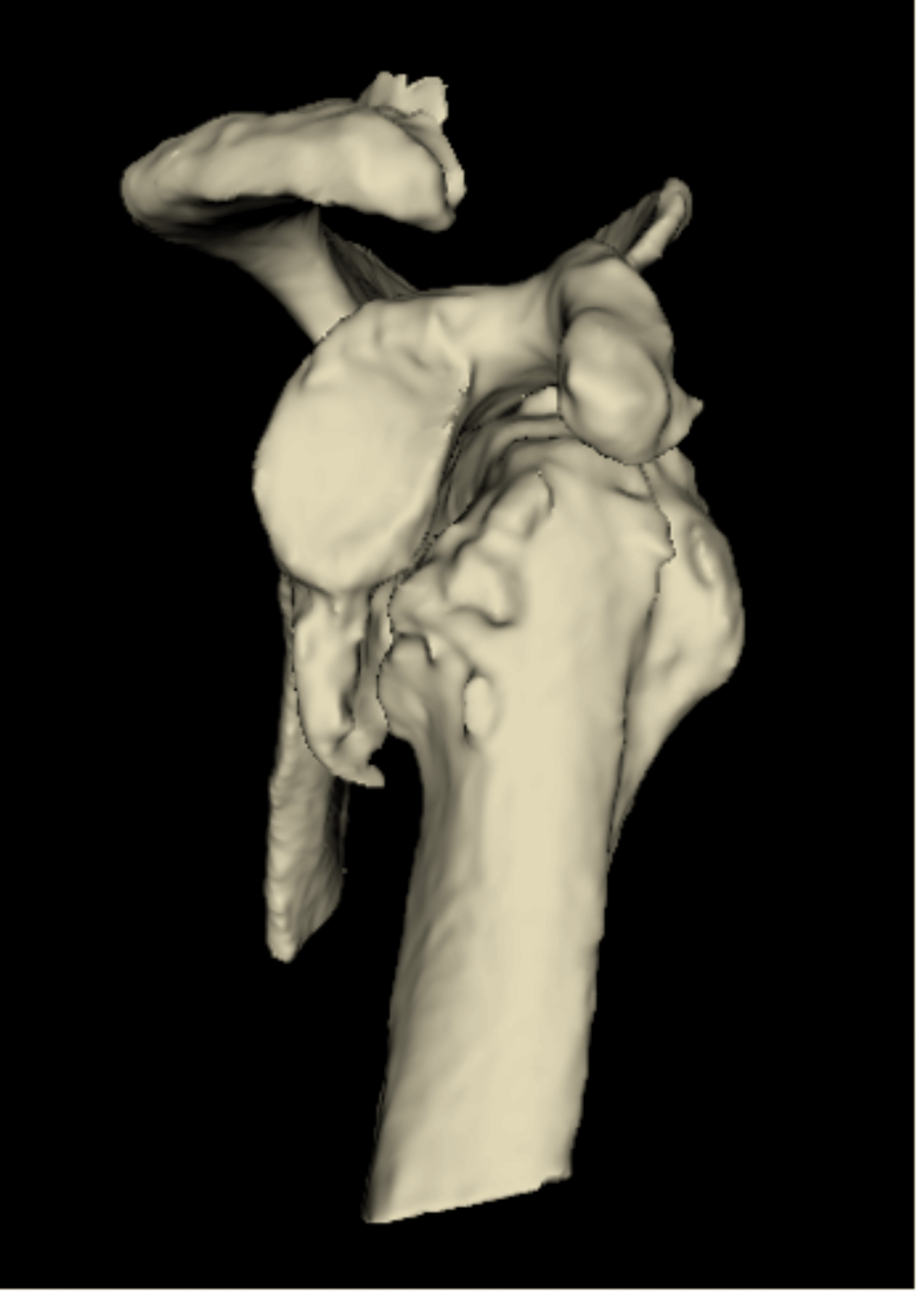
Pre-operative 3D CT sagittal view of the glenohumeral joint demonstrating anterior shoulder dislocation and a glenoid bony defect.

**Figure 3:**
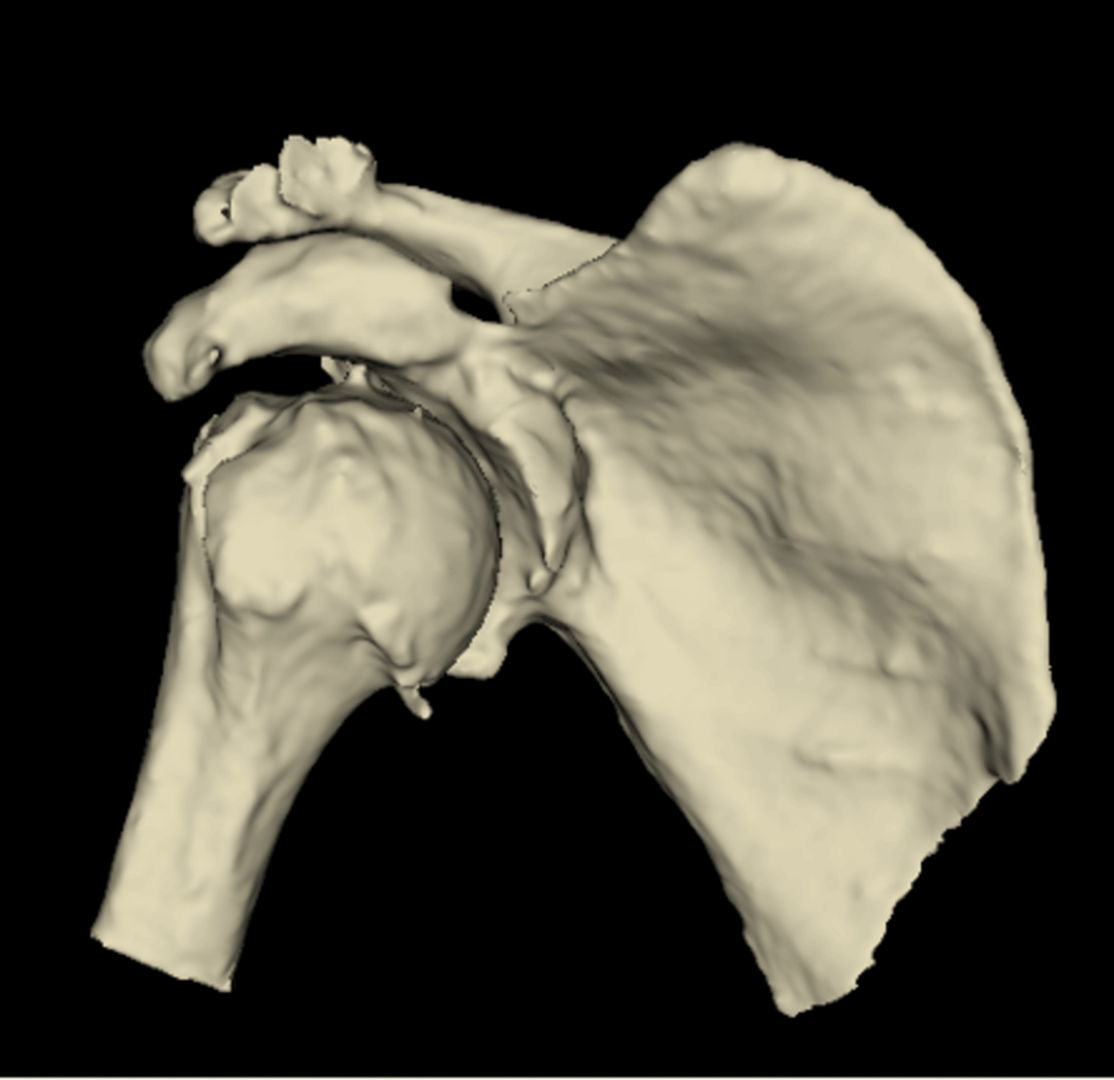
Pre-operative 3D CT coronal view of the glenohumeral joint demonstrating a Hill-Sachs defect.

Treatment options discussed included conservative management, shoulder arthrodesis and reverse shoulder arthroplasty (RSA). Following a thorough discussion with the patient, he opted to proceed with RSA. Computed tomography (CT) further demonstrated anterior shoulder dislocation with a large, 3 cm Hill-Sachs defect and a corresponding defect involving the anterior-inferior bony cortex of the glenoid. New bone formation was noted at the inner aspect of the scapula, just superior to the head of the humerus and inferior to the glenoid (Figures 2, 3). 

**Figure 4:**
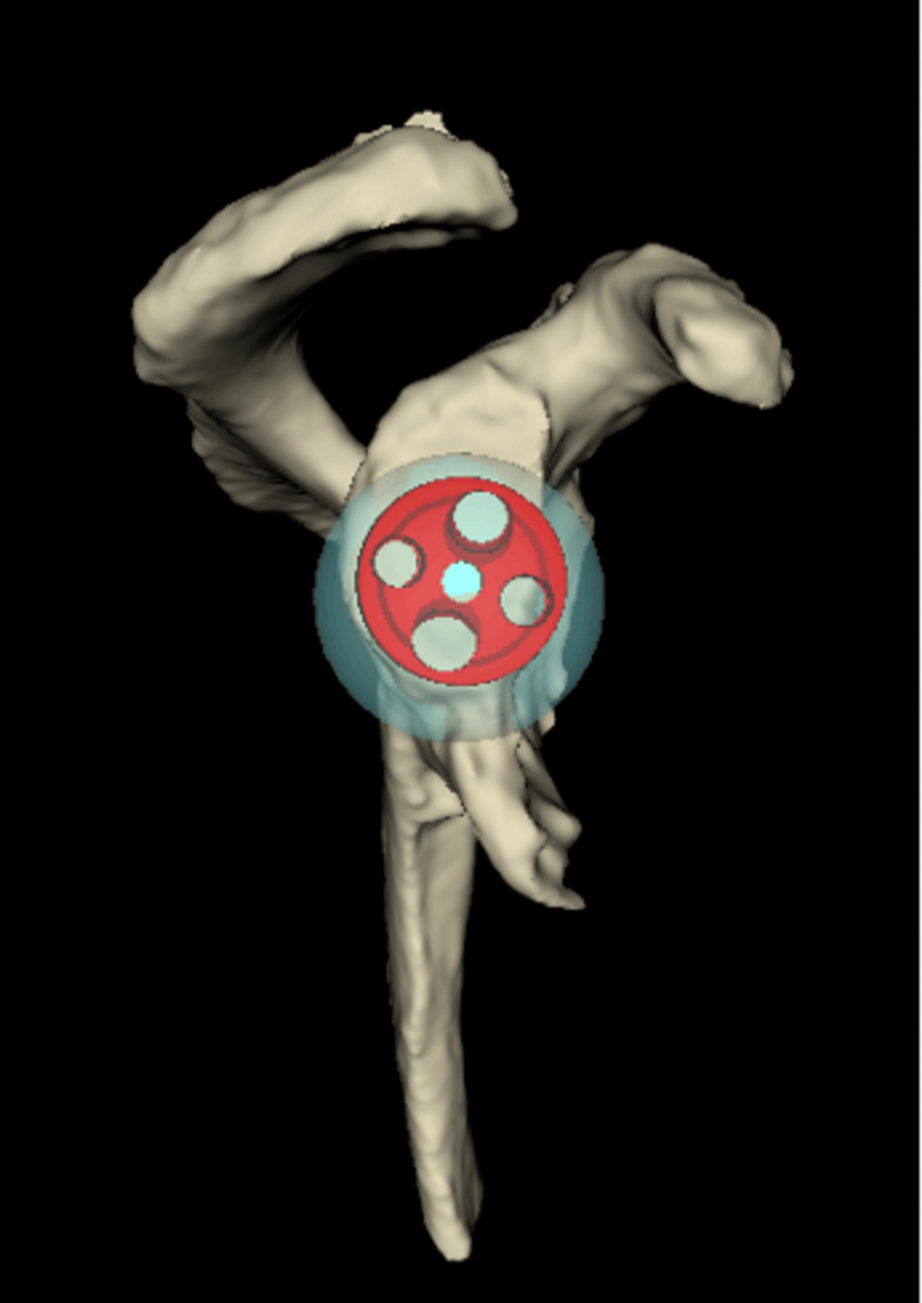
Sagittal 3D CT-based planning view demonstrating planned positioning of a size 24 glenoid baseplate and 36 mm eccentric glenosphere, allowing secure central and peripheral screw fixation despite anterior glenoid bone loss.

**Figure 5:**
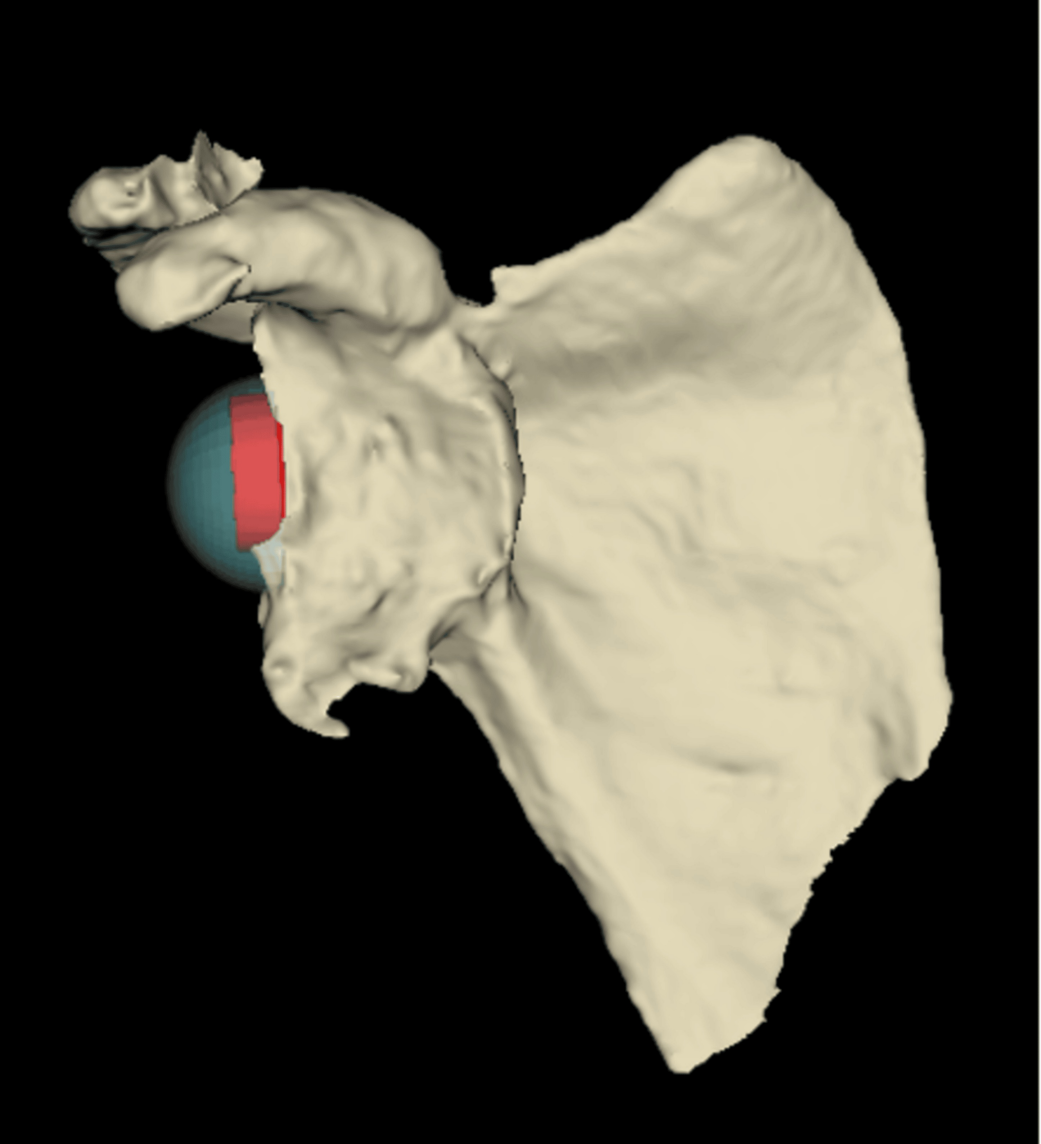
Coronal 3D CT-based planning view demonstrating the glenoid baseplate (size 24) and glenosphere (36 mm).

Pre-operative planning was performed using Blueprint (Wright Medical, Stryker, Kalamazoo, MI, USA) 3D CT-based software, with all data handled in accordance with our institutional governance standards. The pre-operative planning confirmed that no patient-specific instrumentation was needed, whilst a stable glenoid fixation could be accomplished without glenoid structural bone grafting. A 10° clockwise rotation of the glenoid baseplate allowed optimal positioning of a central 6.5 mm screw, two peripheral locking screws (superior and inferior) and one non-locking posterior screw (Figures 4, 5). 

**Figure 6:**
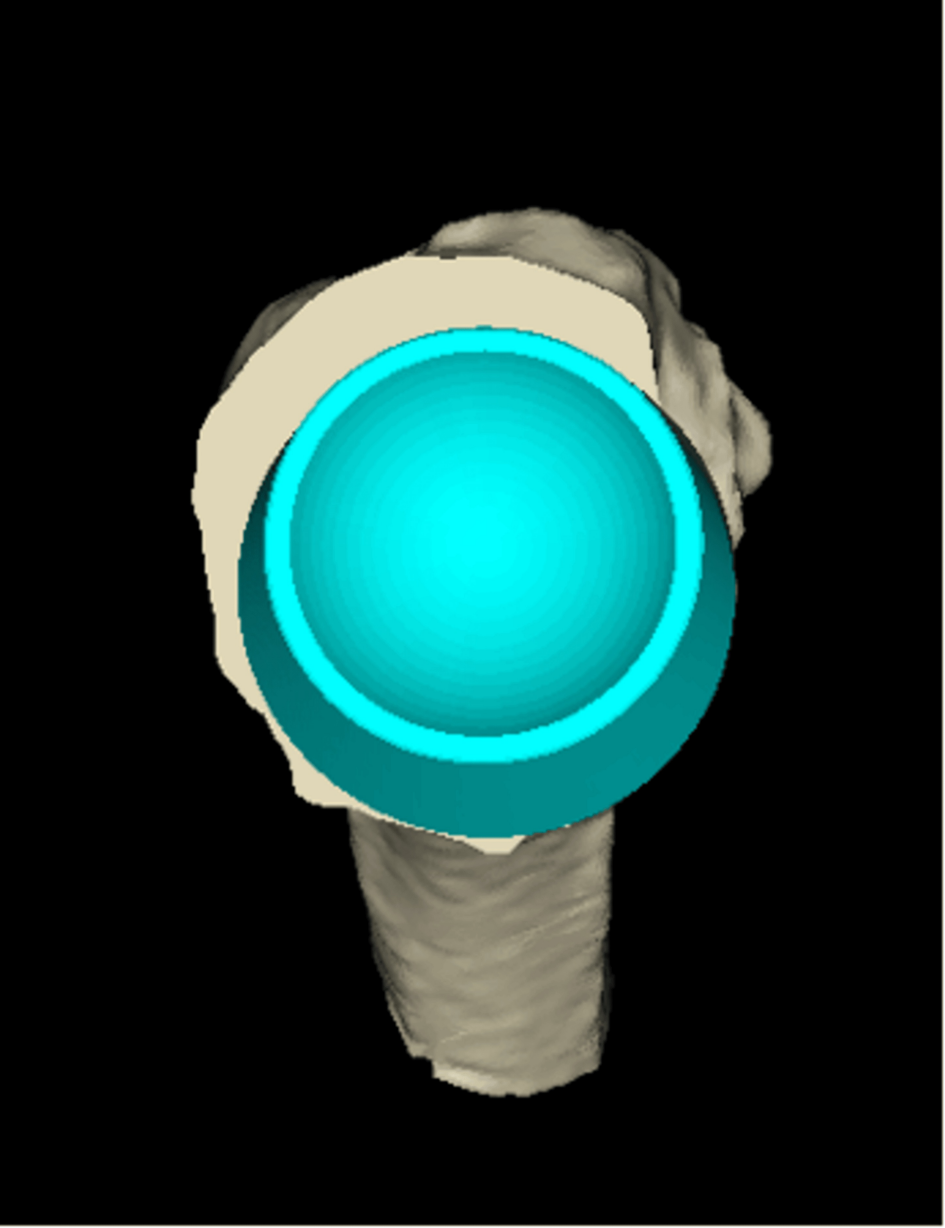
Sagittal 3D CT-based planning view demonstrating the humerus with a high-offset tray and +6 mm liner. The posterior positioning of the offset is shown, designed to counteract anterior soft-tissue tension and enhance joint stability in the setting of chronic anterior dislocation.

**Figure 7:**
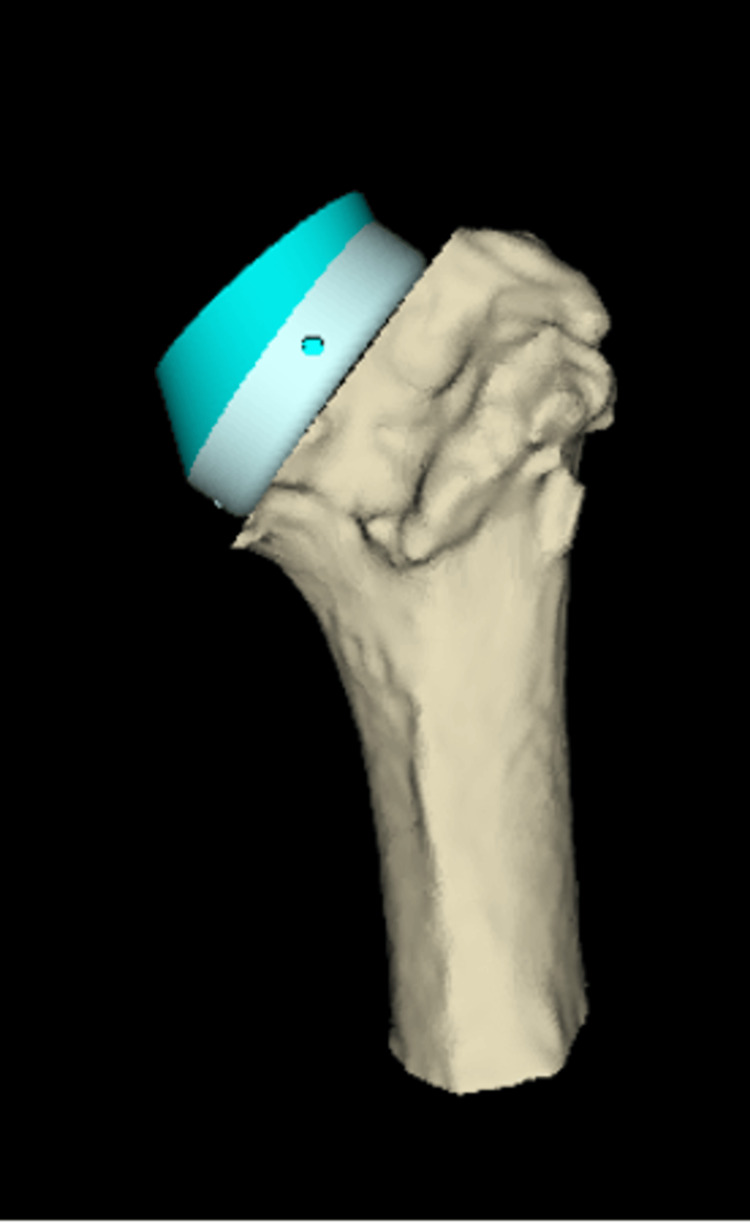
Coronal 3D CT-based planning view demonstrating the humerus with a high-offset tray and +6 mm liner. The configuration demonstrates restoration of humeral length and lateralisation, contributing to improved deltoid tensioning and joint stability.

Given the chronicity of the dislocation (18 months), severe soft-tissue tension was anticipated despite the surgical releases that were planned preoperatively. Therefore, the surgical plan included the use of an eccentric glenosphere (usually with the eccentric part placed inferiorly to prevent glenoid notching on a superiorly placed baseplate) with the eccentric part positioned anteriorly and the use of a high-offset humeral tray with the high-offset positioned posteriorly. This construct was designed to counteract and neutralise the significant forces from the soft-tissue scarring that would force the humeral head anteriorly and make the replacement prone to dislocation and failure. Based on the software, the plan was to use an Ascend Flex stem size 4, size +0 high-offset humeral tray, 36+6 mm humeral insert, perform standard baseplate size 24 mm with a 6.5 mm central screw and an eccentric (+2 mm) 36 mm glenosphere (Figures 4-7). 

On the day of surgery, under a general anaesthetic with an interscalene block, the patient was placed in a semi-inclined chair position at 50°. A standard deltopectoral approach was utilised. Given the position of the humeral head medially and posteriorly to the coracoid, it was deemed unsafe to access the humeral head through a standard subscapularis tenotomy or anterior capsulotomy. Hence, the area of the Hill-Sachs lesion was incised, and the humeral head was approached posterolaterally. The rotator cuff tendons were absent except for the teres minor.

Severe adhesions were found anchoring the humeral head onto the neo-glenoid, which was formed inferiorly and medially to the coracoid. Care was taken with careful dissection within the confinements of the neo-capsule by always staying on the bone. Further to that, a release of the pectoralis major superior tendon at its humeral insertion for about 1 cm was performed while the humerus was released as needed inferiorly to the humeral head. The humeral head was successfully mobilised laterally to the conjoint tendon and then externally rotated.

Humeral head osteotomy was performed with 20° retroversion utilising a forearm reference guide in order to achieve optimal post-operative range of movement without compromising stability.

The humeral shaft was reamed and protected whilst the glenoid was prepared. The glenoid labrum was excised, and a guide pin was applied with the guide. The glenoid was reamed to bleeding bone with the central post drilled as per standard operative technique. The glenoid baseplate was implanted as per the pre-operative planning with only three screws applied, two locking superiorly and inferiorly and one non-locking screw posteriorly due to the bone loss anteriorly. An eccentric glenosphere with the eccentric part positioned anteriorly was applied securely. The definitive humeral stem was then applied with a high-offset humeral tray with the high-offset positioned posteriorly and a +6 mm insert applied on top. The joint was reduced, and it was stable when tested intra-operatively. The construct was washed out, and haemostasis was achieved. The wound was closed in layers. A dressing was applied, and the arm was kept in a polysling.

**Figure 8:**
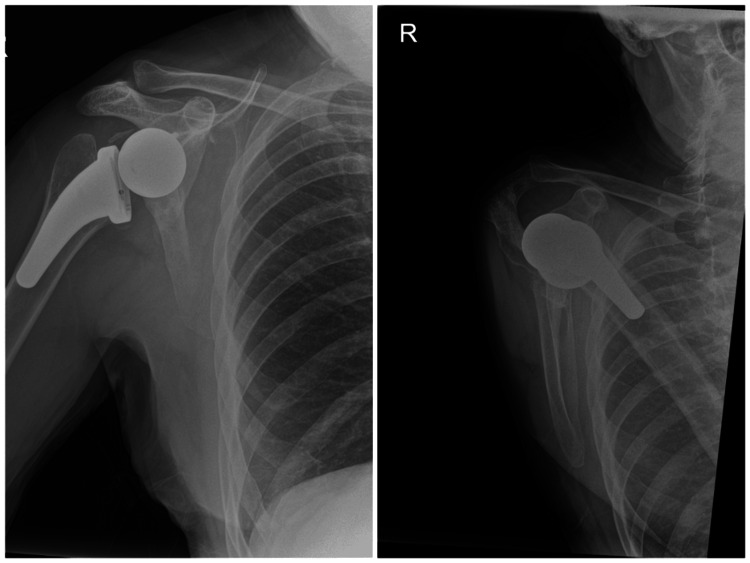
Day-one post-operative radiographs demonstrating well-positioned reverse shoulder arthroplasty with satisfactory implant alignment and stable fixation of the glenoid and humeral components.

Immediate post-operative recovery was unremarkable, and the patient was stable overnight. He received two doses of post-operative teicoplanin as per hospital protocol, and his post-operative bloods and X-ray were satisfactory (Figure 8). He was kept in the polysling with non-weight-bearing advice for six weeks but encouraged to maintain wrist and elbow range of movement. He was reviewed by our physiotherapy team as an inpatient and discharged with exercises. His wound was checked at two weeks with no complications. 

At two months post-procedure, he was pain-free, neurovascularly intact, and his wounds had healed. He achieved 30° of forward flexion and abduction with neutral external rotation.

At four months post-procedure, he remained pain-free with 40° forward flexion, 60° abduction, 30° external rotation and internal rotation with the thumb up to the lumbar spine. He was pleased with the outcome.

He was reviewed at 18 months post-operatively, and he could achieve forward flexion to 130 degrees, abduction to 140 degrees, external rotation to 70 degrees, and his internal rotation to the lumbar spine; his Oxford Shoulder Score was 47/48.

**Figure 9:**
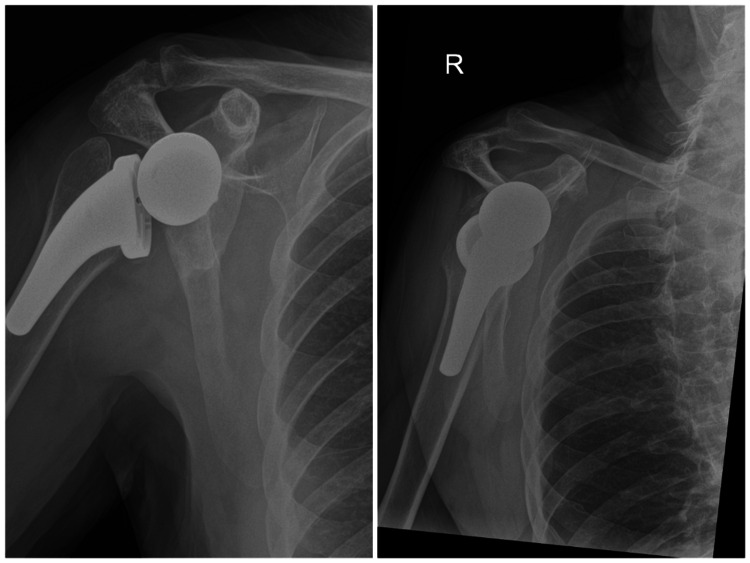
Four-year post-operative radiographs demonstrating maintained implant position with no radiographic evidence of loosening, migration or failure.

Due to the COVID-19 pandemic, there was a delay in further follow-up, and hence he was reviewed again at four years post-operatively, with an X-ray on arrival (Figure 9). Range of motion was forward flexion and abduction to 140°, internal rotation to the lumbar spine, and external rotation to 30° with no obvious clinical or radiographic signs of impingement. Despite his reduction in range of motion since his last review, his Oxford Shoulder Score was 48/48.

## Discussion

Anterior shoulder dislocations are common orthopaedic injuries and affect mainly young men and the elderly [4]. In contrast, chronic locked anterior shoulder dislocations are rarer occurrences and can present a surgical challenge due to humeral and glenoid bone loss.

Owing to the limited number of reported cases, there is no consensus on the optimal management of chronic anterior dislocations. Described techniques range from stabilisation procedures such as coracoid osteotomy or Bristow-Latarjet procedure to arthroplasty options including humeral head resurfacing, hemiarthroplasty, reverse shoulder arthroplasty and total shoulder arthroplasty [5-9]. In patients younger than 45 years, Sahu et al. reported poor functional outcomes with conservative management and open reduction techniques, recommending preservation of the subscapularis and reconstruction of the anterior glenoid deficiency using bone graft [10].

The choice of treatment depends on patient factors, such as age and functional level, as well as the integrity of the rotator cuff and glenohumeral joint condition. For patients with irreparable rotator cuff tears and fixed anterior dislocation, treatment with hemiarthroplasty is not recommended, and the RSA is the more preferred option [11].

Bony defects associated with recurrent anterior glenohumeral instability have been reported in 18% of glenoids, 30% of humeral heads and both in 22% of shoulders [3]. These defects present a challenge to the surgeon. Management strategies include eccentric reaming, glenoid bone grafting and augmented glenoid components. Eccentric reaming risks excessive medialization and reduced soft-tissue tension, increasing the risk of instability. In contrast, bone grafting of the glenoid defect in primary RSA can restore version and preserve bone stock [12].

A recent meta-analysis demonstrated high rates of graft union, low rates of scapular notching and acceptable complication and revision rates [13]. However, lab-based studies suggest that glenoid bone coverage of 50% or more is sufficient for stable fixation and osseous integration even without bone grafting, where glenoid bone loss is present [14].

In this case, 3D CT-based Blueprint planning enabled us to have an accurate characterisation of the glenoid as well as to perform a virtual implantation and trial without error. By using the 3D CT-based planning software, it was identified that the patient’s glenoid bony defect was classified as type 3A: anterior glenoid deficiency <25%, according to the Bigliani classification [15]. Based on preserved bone stock, a decision was made to opt against bone grafting or augmented components. A trabecular metal baseplate was selected for improved osseointegration.

The fixation of the baseplate with one central and three peripheral screws was deemed a safer option. Furthermore, an implant (screw or tape) for graft fixation could cause non-optimal baseplate screw positioning, trying to bypass with the baseplate screws. It would not add to the stability since it would be technically challenging, if impossible, to utilise the fourth peripheral baseplate screw through the graft. Although a recent systematic review supports excellent outcomes with primary RSA and bone grafting, augmentation was not needed in this case, given the absence of significant concentric or eccentric glenoid erosion secondary to chronic dislocation [16].

This case report is descriptive in nature and reflects a technical approach rather than a comparative study. The described workflow relies on access to 3D CT-based planning software and accurate image segmentation, which may not be universally available. The proposed planning strategy is specific to chronic anterior shoulder dislocation and may not be directly applicable to other patterns of instability. 

## Conclusions

This case highlights the complexity of managing chronic anterior shoulder dislocations and underscores the value of pre-operative planning. In the presence of an 18-month fixed dislocation, anterior glenoid bone loss and severe soft-tissue contracture, RSA proved to be a reliable and effective treatment option. Three-dimensional CT-based planning allowed precise characterisation of the glenoid defect and informed intraoperative decision-making, enabling secure baseplate fixation without the need for structural bone grafting. Strategic orientation of the eccentric glenosphere and high-offset humeral tray effectively counteracted chronic soft-tissue forces, contributing to a stable construct.

Despite the chronicity of the injury and the technical challenges of the procedure, the patient achieved excellent functional outcomes, maintaining an Oxford Shoulder Score of 48/48. Reverse shoulder arthroplasty remains a viable option for similar patients, particularly those with rotator cuff arthropathy, bone loss, or mobility requirements that necessitate reliable upper-limb function.
